# Automated 3-D Computer-Aided Measurement of the Bony Orbit: Evaluation of Correlations among Volume, Depth, and Surface Area

**DOI:** 10.3390/jpm14050508

**Published:** 2024-05-11

**Authors:** Risto Kontio, Tommy Wilkman, Karri Mesimäki, Yurii Chepurnyi, Antti Asikainen, Aleksi Haapanen, Arto Poutala, Marko Mikkonen, Alina Slobodianiuk, Andrii Kopchak

**Affiliations:** 1 Department of Oral and Maxillofacial Surgery, Helsinki University Hospital, 00290 Helsinki, Finland; risto.kontio@peakan.fi (R.K.); tommy.wilkman@hus.fi (T.W.); karri.mesimaki@hus.fi (K.M.); asikainen@luke.fi (A.A.); aleksi.haapanen@helsinki.fi (A.H.); 2 Institute of Oral and Maxillofacial Diseases, Helsinki University, 00014 Helsinki, Finland; 3 Department of Maxillofacial Surgery and Modern Dental Technologies, O.O.Bogomolets Medical University, 02000 Kyiv, Ukraine; 80667788837@ukr.net (Y.C.); kopchak@ua.fm (A.K.); 4 Disior, Maria 01, Building 2, Lapinlahdenkatu 16, 00180 Helsinki, Finland; arto@disior.com (A.P.); marko@disior.com (M.M.)

**Keywords:** orbit, orbital volume, automated CT segmentation, orbital depth

## Abstract

(1)The study aimed to measure the depth, volume, and surface area of the intact human orbit by applying an automated method of CT segmentation and to evaluate correlations among depth, volume, and surface area. Additionally, the relative increases in volume and surface area in proportion to the diagonal of the orbit were assessed. (2) CT data from 174 patients were analyzed. A ball-shaped mesh consisting of tetrahedral elements was inserted inside orbits until it encountered the bony boundaries. Orbital volume, area depth, and their correlations were measured. For the validation, an ICC was used. (3) The differences between genders were significant (*p* < 10^−7^) but there were no differences between sides. When comparing orbit from larger to smaller, a paired sample *t*-test indicated a significant difference in groups (*p* < 10^−10^). A simple linear model (Volume~1 + Gender + Depth + Gender:Depth) revealed that only depth had a significant effect on volume (*p* < 10^−19^). The ICCs were 1.0. (4) Orbital volume, depth, and surface area measurements based on an automated CT segmentation algorithm demonstrated high repeatability and reliability. Male orbits were always larger on average by 14%. There were no differences between the sides. The volume and surface area ratio did not differ between genders and was approximately 0.75.

## 1. Introduction

Orbital volume is one of the most important parameters in orbital surgery, which has been noted by numerous studies [1,2,3,4,5]. Additionally, variations in volume, surface area, and locations of orbit fractures are reported to be crucial factors in surgical decision making [6,7,8,9]. These factors are applied to describe the severity of trauma and the efficacy of orbital reconstruction. However, different methods of measurement, both manual and digital, based on CT may yield different absolute values.

Accurate measurement of the orbit has proven to be technically challenging. In a retrospective study by Ozdikici et al. [10] with a total of 302 adults, the average right and left orbit (including eyeball) depth was 52 mm for men and 50 mm for women. Numerous studies have confirmed that the average orbit volume is between 23 mL and 30 mL [11,12]. In a study by Deveci and coworkers [13], the mean volume was 28.37 mL ± 2.15 mL by the direct impression technique and 28.41 mL ± 2.09 mL by CT-based software measurement. This difference between CT-based and manually measured orbital volumes has been documented by additional studies. This necessitates the examination of correlations among some geometric parameters of the orbit. These correlations could be applied to evaluate orbital trauma severity, make decisions, and create a statistical shape model. Therefore, the dimensions of the bony orbit are crucially important.

Despite difficulties related to the complicated procedure of manual CT segmentation, CT has become the standard practice in orbit assessment because of its easy access and high resolution. The time-consuming nature of this procedure is recognized as its main drawback. However, due to the development of new automated algorithms, orbital depth and volume may be measured with the same accuracy and comparable speed [14,15]. CT-based measurements are, with 95% likelihood, within −1.8 mL and +2.6 mL of the correct volume [16]. The main reason for this, indicated in numerous studies, is the method for determining the orbital anterior closure [17,18]. Volume differences (volume ratio), although defined differently, could similarly be independent of the methodology of measurement. This is true for the volume ratio measured by different methods of CT segmentation [14]. Orbital volume differences between right and left or injured/reconstructed and intact orbits and volume–depth correlations would solve the problem of determining orbital anterior closure and permit the comparison of results achieved via different (manual, semi-automated, or automated) methods of CT segmentation.

Numerous studies have demonstrated the efficacy of the mirroring technique (including in automated mode) in orbital reconstruction involving the application of patient-specific implants [19,20]. However, slight differences in volume between the right and left orbit were documented in a study of human skulls [21]. The difference between left and right orbit volume was on average 0.8 mL, with volumes of 26.7 mL for the left and 27.5 mL for the right. Approximately 14% of skulls showed left–right orbit volume differences of 1.5 mL or greater and 21% showed differences of 1.0 mL or greater [21]. In contrast, Ozdikici et al. [10] defined the average volume of the orbit as 25 mL for women and 29 mL for men and noted the absence of significant differences between the left and right orbits.

However, other three-dimensional orbital measurements are still underestimated and have been evaluated by only a few authors. Kang and Han [22], measuring orbit depth, defined the distance from the optic foramen to the orbital rim as 49.60 mm and the distance from the optic foramen to the lacrimal crest as 41.32 mm. Felding et al. [11] documented a mean surface area of the orbits of human cadavers of 32.47 cm^2^ ± 2.96 cm^2^; the average difference between the left and right orbits was 4.1% and was not significant.

At the same time, there are several studies in the literature devoted to an automated algorithm measuring the orbital volume with small samples. The results obtained by the authors [19,23] showed high repeatability of reproducibility of the automatic method, which prompted us to conduct this study.

The primary aims of the present study were to measure the depth, total volume, and surface area of the intact human orbit through the application of an automated method of CT segmentation and to evaluate correlations among depth, volume, and surface area. Additionally, the relative increases in volume and surface area in proportion to the diagonal of the orbit were assessed.

## 2. Materials and Methods

CT data from 202 patients who had undergone diagnostic procedures at the Center of Maxillofacial Surgery and Stomatology of Kyiv Regional Hospital were analyzed. CT procedures were performed according to a standardized diagnostic protocol. Data were obtained from two CT machines: Philips Brilliance iCT 128TM (0.68 mm slice thickness, 120 kV, 300 mA, 512 × 512 image matrix) and GE Revolution EVO 128TM (0.68 mm slice thickness, 80 kV, 500 mA, 512 × 512 image matrix). Radiological inclusion criteria were the following: both orbits intact, no radiological symptoms of bony orbit deficiency, no visible pathology of the middle face, and patients older than 18 years and with information on age and gender.

**Figure 1:**
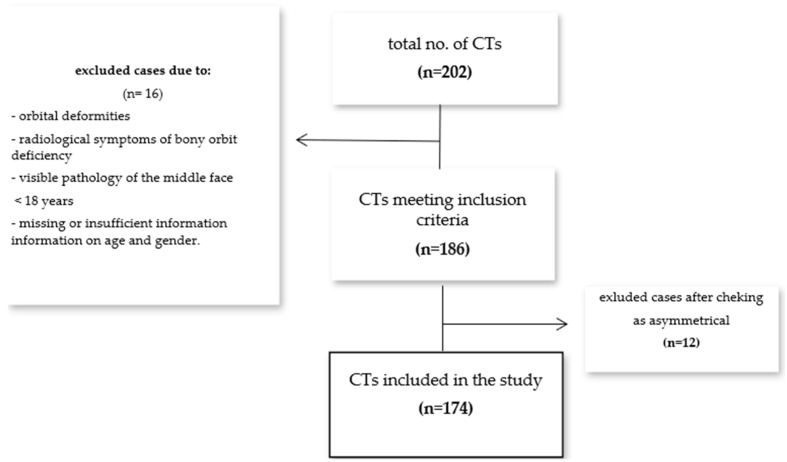
Patient inclusion flowchart.

CT data from 186 patients who met the inclusion criteria were imported into the software. In the first phase, the software algorithm compared the orbits of each patient. The orbits were determined to be asymmetric if they differed in volume by more than 0.2 mL. Four men and eight women were identified as having asymmetric orbits. Asymmetric orbits were excluded and analyzed manually (Figure 1). 

CT data from 174 patients were then included in the study. These patients included 91 men and 83 women with a mean age of 38.49 ± 13.52 years (range 18–74). CT images were imported in the DICOM format into the Disior CMF Orbital 2.2.2021 analysis software (Helsinki, Finland). After import, Disior automatically converted the image information into a voxel map. The bone threshold value was then defined by automated algorithms from the image data. The value was optimized to find the best descriptive HU to define the bone structures. Based on the voxel data, a mesh consisting of tetrahedral elements was used to model the orbital shape and volume. Special care was taken to handle the extremely thin bone lining, which is usually non-continuous in CT images because of the slice interval, slice thickness, and CT voxel size.

**Figure 2:**
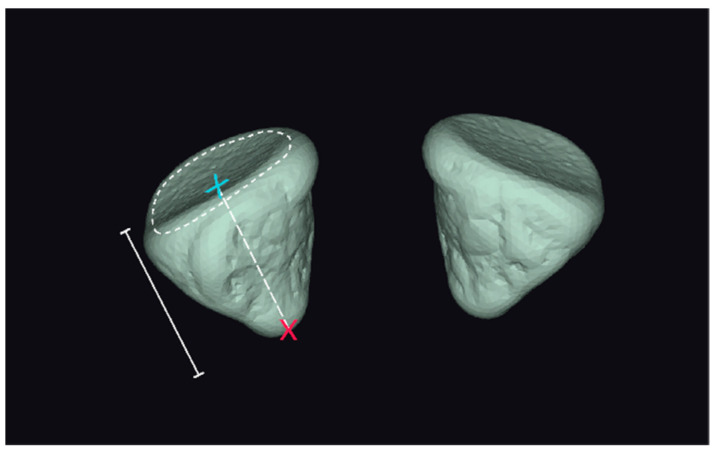
Axial orbital length is the distance between the posterior point of the orbital volume (red ‘x’) to the mid-point (blue ‘x’) of the anterior closing surface (‘----’).

Anterior and posterior closures were defined automatically. As a result, the following characteristics were assessed as orbit dimensions:

(1) Volumes of left- and right-side orbits (meshes): The volumes of the left- and right-side meshes were calculated as the sum of the volumes of the tetrahedral elements that resulted from the segmentation algorithm;
(2) Surface areas of left- and right-side orbits (meshes): The surface areas of the left- and right-side meshes were calculated as the sum of the areas of the surface elements of the orbit;
(3) Depth measurement of the orbit: This measurement is visualized in Figure 2.

**Figure 3:**
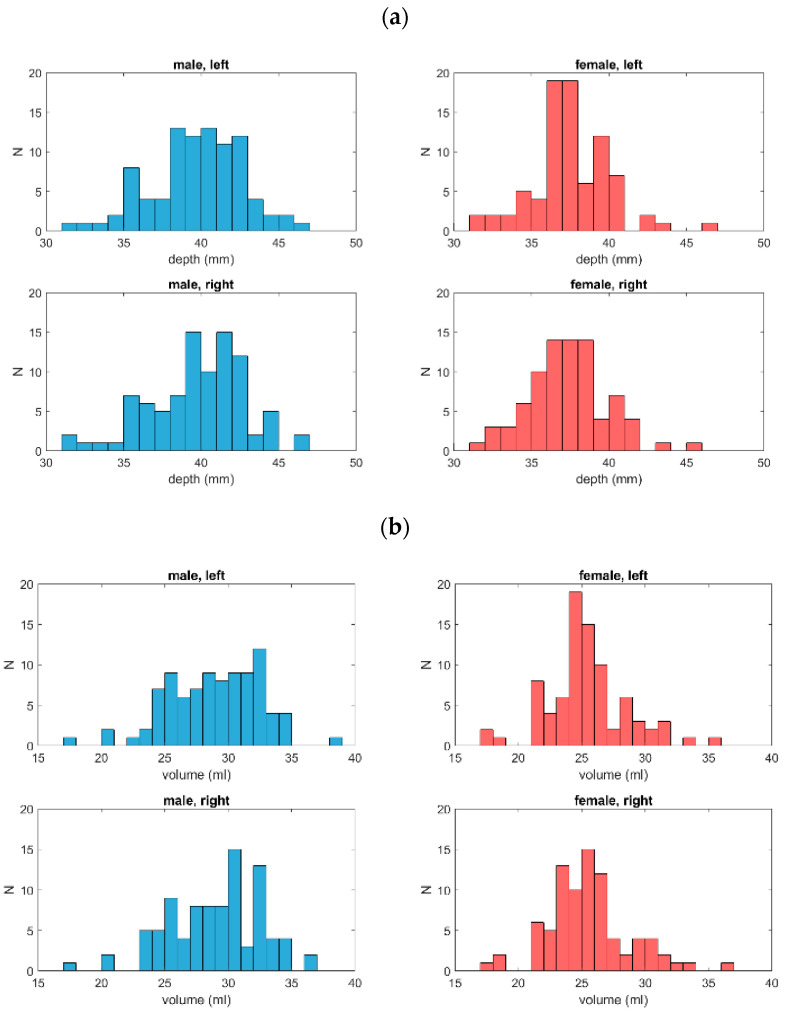

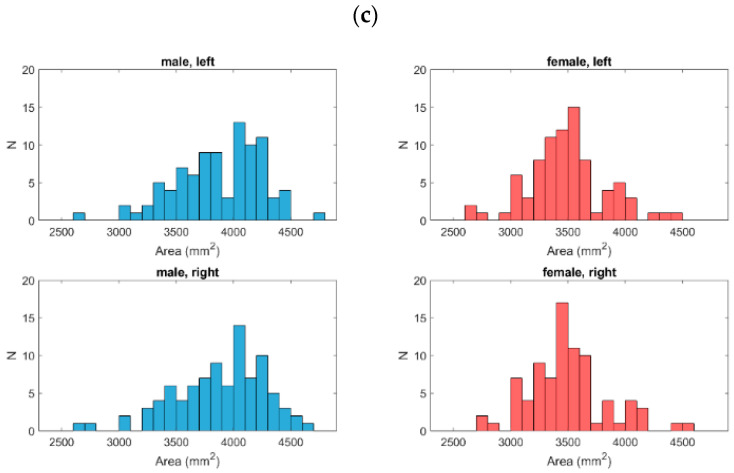
Distributions of orbital depth (**a**), orbital volume (**b**), and orbital surface area (**c**) for both groups and both orbits. For depth and volume, the data were normally distributed in the male group and in the right orbit of the female group. In addition, the distribution of surface areas for the left orbit of the male group was normally distributed.

Volume is presented as a function of the orbit depth normalized to a scale of 0–10. This was achieved by dividing the depth axis into 10 equally long pieces, projecting the centers of each tetrahedron of the segmented model to this axis, and calculating the sum of volumes for those tetrahedra that were projected on the piece. Any volume anterior to the mid-point of the anterior closure surface was excluded (Figure 3).

For the validation, CT data from 10 patients were randomly selected from the data for the 174 patients. Six maxillofacial surgeons individually imported the 10 datasets into the software twice. The software was turned off between each validation. Each orbit was measured 12 times, for a total of 240 measurements. The volumes were measured automatically. Intra- and interobserver differences were evaluated using intraclass correlation coefficients (ICCs). MATLAB (version 2020b, Mathworks Inc., Natick, MA, USA) was used for the analysis.

## 3. Results

We assessed 174 pairs of orbits (91 men and 83 women). Based on a Shapiro–Wilk test, both the depth and the volume were normally distributed in the male group and in the right orbit of the female group. In addition, the distribution of surface areas for the left orbit of the male group was normally distributed (Figure 3a–c; Table 1).

Average orbit depth, volume, and surface area, as well as their standard deviations and ranges for both the male and female groups and for both orbits, are presented in Table 2.

For all three parameters, the differences between the male and female groups were significant (*p* < 10^−7^) but no significant differences between left and right orbits were found in either group (*p* > 0.09). However, when comparing the larger orbit to a smaller one, a paired samples *t*-test indicated a significant difference in both groups and all three parameters (*p* < 10^−10^). In contrast, when the individual orbits were considered as independent samples, no significant difference between larger and smaller orbits was found in any of the aforementioned cases (*p* > 0.15). The individual *p*-values are reported in Table 3.

**Figure 4:**
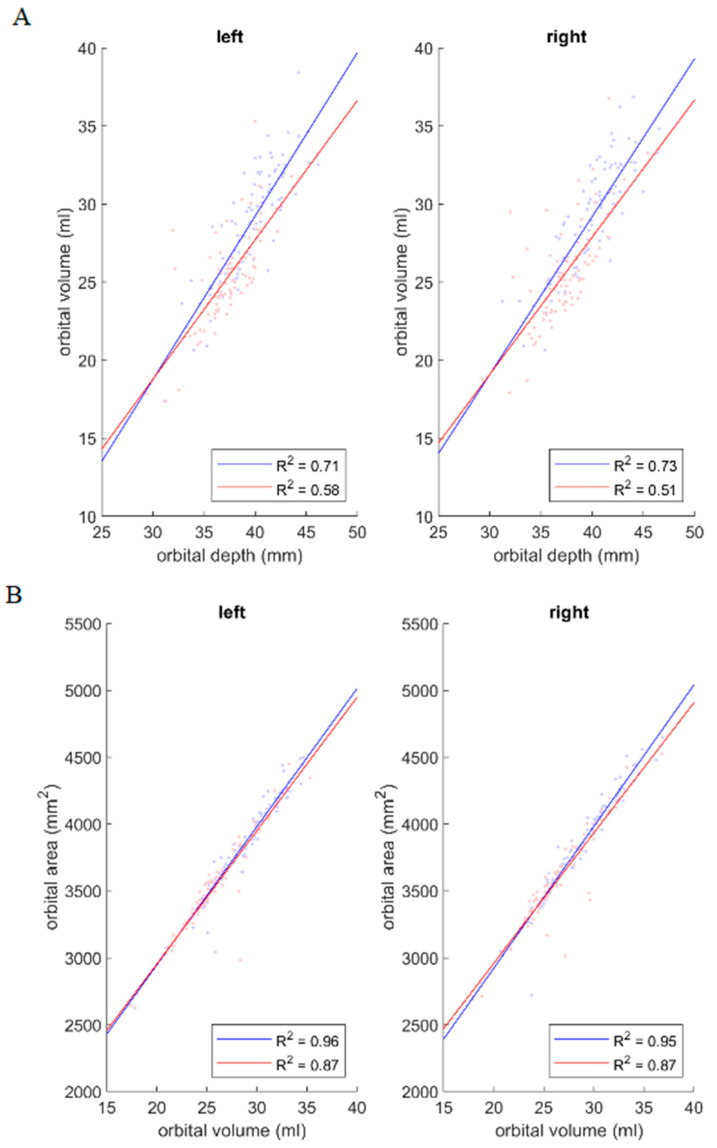

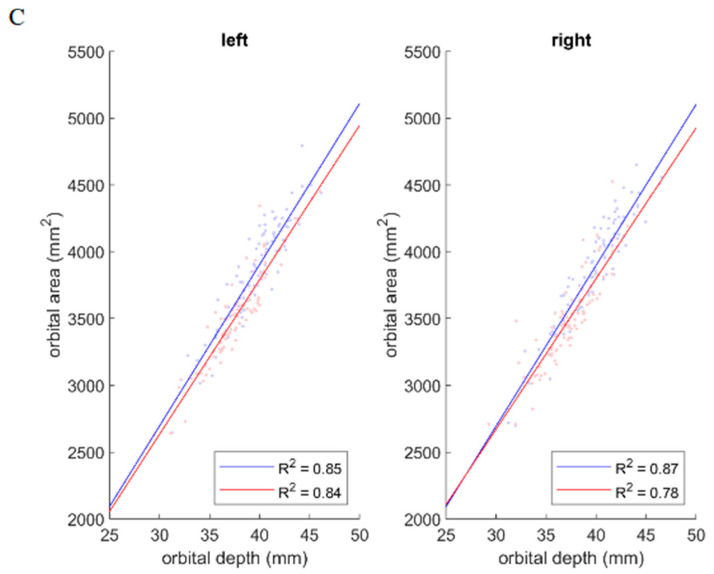
(**A**) Orbital volume as a function of orbital depth, (**B**) orbital surface area as a function of orbital volume, and (**C**) orbital surface area as a function of orbital length. In each pair, a positive correlation was observed between the parameters in both male (blue) and female (red) groups and in both orbits. Correlation coefficients (R^2^) for each case are reported in the figure.

Correlations among depth, volume, and surface area are presented in Table 4 and Figure 4A–C.

Strong positive correlations were observed between orbit depth and surface area and between orbit volume and surface area in both gender groups and for both orbits. A strong positive correlation was also identified between orbit volume and depth for both orbits in the male group, whereas this correlation was only moderate for both orbits in the female group (Figure 4).

As there was a slight difference in the strength of the correlation between orbit volume and depth between the male and female groups, the effect of gender was assessed using a simple linear model (Volume~1 + Gender + Depth + Gender:Depth). This revealed that only depth had a significant effect on volume (*p* < 10^−19^). Gender and the interaction of gender and depth were not significant predictors of volume (*p* > 0.17) (Table 5).

**Figure 5:**
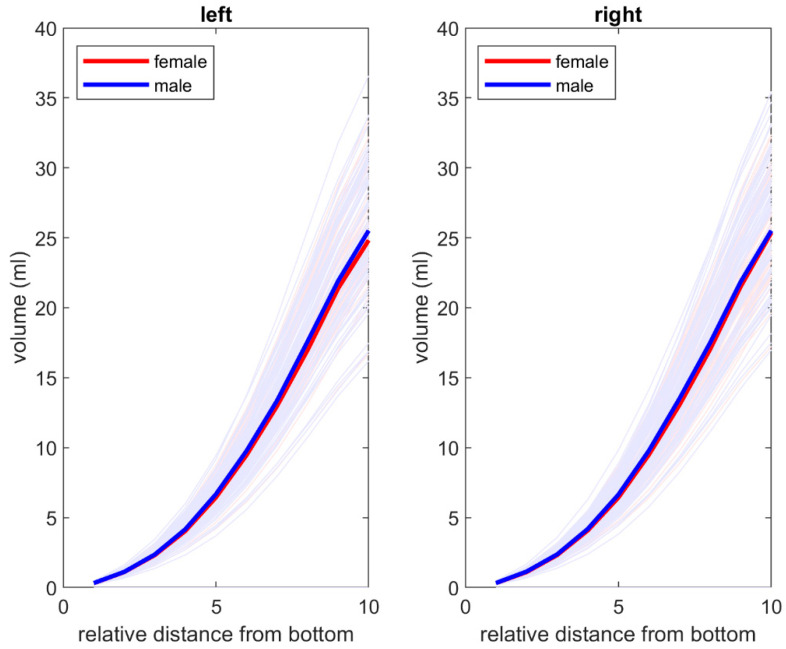
Volume as a function of relative distance from the apex for male (blue) and female (red) groups for **left** and **right** orbit respectively. The volume increases in a similar manner in both groups and both orbits.

Additionally, an increase in volume as a function of the relative distance from the apex was observed and was similar in men and women. The data are presented in Figure 5.

**Figure 6:**
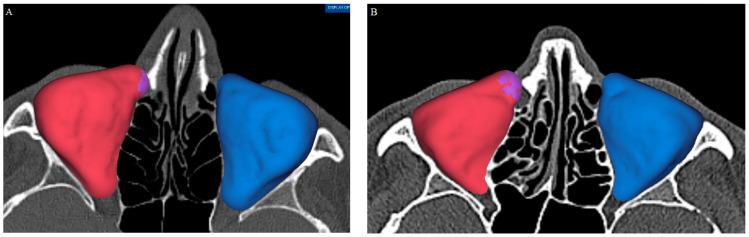
Mucosal thickening of the lacrimal duct resulted in the lacrimal duct being included in the automated segmentation of the orbit, leading to an overestimation of the orbital dimensions. Two examples are presented: (**A**) and (**B**). These cases were excluded from the study.

The 12 patients (4 men and 8 women) with asymmetric orbits were examined in detail using original CT data. In five cases, mucosal thickening of the lacrimal duct was discovered and resulted in an error in automated segmentation (Figure 6A,B).

In three cases, a fractured orbit was identified. In four cases, accounting for only 1.98% of the evaluated population, generalized asymmetry of the face or frontal area was detected. These 12 patients were excluded from statistical analyses.

For validation, intra- and interobserver differences in volume measurements were analyzed. The ICCs for the intraobserver and interobserver agreement of volume measurements were excellent: both were 1.0. This confirms that the automated algorithm assessment of the images was repeatable. The data are presented in Table 6.

## 4. Discussion

The application and testing of automated algorithms for CT segmentation have been recent directions of scientific research, including on maxillofacial surgery [14,15,24]. This automated method aims to permit a less time-consuming procedure with the same accuracy and reproducibility as manual measurement. The automated method is especially helpful for orbital surgery due to the orbit’s size and complex anatomy. Not only does the orbit have an imprecise pyramidal form with thin walls that are often not displayed as a solid surface on CT images but it also lacks an anterior border and has several posterior anatomical gaps.

Jansen and coworkers have studied automated software segmentation. They focused on the accuracy and reproducibility of measurements of the volume of the intact bony orbit [19]. The automated segmentation method used the subtraction of bone and air density masks. The semi-automated method also used manual adjustment. The automated segmentation proved to be superior and more repeatable than the semi-automated method. The authors therefore highly recommended automated segmentation [23]. In accordance with other studies, our results demonstrated the possibilities of automated CT segmentation for the estimation of important orbital measurements, such as orbital volume, volume differences, depth, surface area, and their correlations. An estimated automated algorithm produced identical results in repetitive testing sessions and with several individual users [12,15,22]. Based on good accuracy and repeatability, this made it possible to detect volume differences of 0.2 mL [16,25]. As a result, only approximately 2% of patients of both genders were recognized as having asymmetric orbits without any known reasons in the present study. However, we noticed that anatomic aberrations may result in error. In five cases, the mucosal thickening of the lacrimal duct opening resulted in an erroneous volume measurement. Automated methods are accurate at present but still require human control. However, automated methods provide opportunities for follow-up studies due to their repeatability and reproducibility.

Tandon and coworkers [21] used 121 skulls to study orbit volumes. Alginate impressions were taken and the volume of each impression was calculated via the volume-displacement method. The mean right orbit volume was 26.75 cm^3^ ± 3.2 cm^3^ (17.7–34.7 cm^3^). The mean left orbit volume was 26.65 cm^3^ ± 3.2 cm^3^ (16.4–35.5 cm^3^). The right–left orbit volume difference was 0.8 cm^3^. This difference was not statistically significant but the authors concluded that the finding was clinically relevant. Notably, this study identified great variation in orbit volumes [21]. Other studies have produced similar findings [19,26,27].

The results of the present study are consistent with those of other studies of orbit dimensions. We did not find any significant differences between the left and right orbits of individuals but there was a significant difference between genders in depth, volume, and surface area of orbits. Ozdikici et al. [10] conducted a retrospective study of orbit dimensions using CT scans of healthy adults. Their study included 302 adults and 34 parameters were measured for both orbits. The study revealed that men had statistically significantly higher mean values than women (*p* < 0.05). There were no statistically significant differences between the measurements of the right and left orbits (*p* > 0.05). According to the percentiles of measurements of orbit eyeball depths, 95% of females had a depth of 52 mm or less and 95% of males had a depth of 56 mm or less [10]. This is in accordance with our study in which the average depth of the orbit differed significantly between men and women. In our study, the average depths were 40 mm for men and 37 mm for women. The average difference in depth between genders in our study was nearly 8%, similar to the results of Ozdikici et al. [10] acquired by manual segmentation. These results provide a strong foundation for the wide application of mirroring techniques to design patient-specific solutions based on automated CT segmentation for orbital surgery among a Caucasian population.

One interesting finding was the great variation in orbit depths. In men, depths were between 31 and 47 mm and between 29 and 46 mm in women. Although there were no significant differences between the left and right orbits, there was a significant difference between the shortest and longest orbit depths. Ozdikici et al. [10] conducted a retrospective study of orbit volumes in healthy adults. Female orbit volume was on average 37 cm^3^ and male average volume was 43 cm^3^. Ching and coworkers [28] used 70 randomly selected CT facial scans of adults. Scans were reconstructed in the software package Mimics 18.0. Several parameters were analyzed. No differences were found between the volumes of orbits of each individual. Males had an orbit volume of 3.07 cm^3^ larger on average than females (29.58 cm^3^ versus 26.51 cm^3^; *p* = 0.0002). This may have great clinical importance but requires further clinical studies. The evaluation of orbital volume diversity within genders can explain the univariate correlation between the size of bone defects and the severity of exophthalmos after orbital trauma [7,8]. There is a significant difference between genders in orbit volume. The absolute values vary between publications but the volume difference between genders seems to be 14% [10,28]. In the present study, the volume difference between genders was also 14%.

Felding et al. [11] estimated the volume and surface area of orbits in CT scans of 11 human cadavers using stereological sampling techniques. CT data files were converted to JPEG and analyzed using ImageJ open-source software, version 1.43. The mean volume and surface area of the orbits were 24.27 cm^3^ ± 3.88 cm^3^ and 32.47 cm^2^ ± 2.96 cm^2^. There were no significant differences in volume (*p* = 0.315) or surface area (*p* = 0.566) among the orbits. Again, notably, there was great variation in orbit volume (19.24–32.03 cm^3^) and in orbit surface area (28.21–38.44 cm^2^). Such variation was also detected by Metzger and coworkers [29] in a topographical study of orbits. Of 279 orbit CT scans, they were able to separate orbits into 12 distinct groups based on variation. In our study, the surface area varied in males from 26 cm^2^ to 48 cm^2^ (mean 39 cm^2^) and in females from 26 cm^2^ to 44 cm^2^ (mean 35 cm^2^). Interestingly, the ratio between volume and surface area was the same between genders and was similar to that found by Felding et al. [11]. This ratio seems to be approximately 0.75.

In our study, great variation in depth, volume, and surface area among individual orbits was detected. Differences were significant. This finding is supported by Metzger and his coworkers [29]. The statistical analysis showed strong correlations among depth, volume, and surface area. There are not many studies available that examine these correlations; however, this is crucially important.

Based on the statistical analysis, only depth had a significant effect on volume. Gender and the interaction of gender and depth of the orbit did not have significant effects on the orbit volume. A similar positive correlation was found between the relative distance from the apex area and the degree of volume increase. It is notable that the graphs are remarkably similar for the right and left orbits and for both genders. Interestingly, the largest increase in volume per unit length of the orbit was found in its anterior third. This part of the orbit is located mostly in front of the ocular globe equator, with the other two-thirds behind it. In addition, the part of the orbital volume behind the ocular globe is responsible for sagittal dislocation caused by different orbital pathologies [7,8,9,30]. The less pronounced volume changes that appeared there resulted in a stronger influence on ocular globe position, compared to the anterior third; this was shown by several clinical studies. This leads to the opposite assumption: the same volume differences behind the ocular globe can cause different dislocations of the ocular globe in individuals with different orbital depths. Therefore, the correlations among eyeball position, volume behind the ocular globe, and orbital depth require further and deeper investigation.

Although the results are very preliminary and require further research, this technique could be applied as a universal method of orbital anatomy evaluation not only for orbital trauma management but also in the treatment of orbital neoplasms and autoimmune diseases, such as Graves’ disease or pseudotumor, accompanied by orbital wall defects and deformities. The application of the proposed method of automatic orbital segmentation can be used to determine the extent of asymmetry as a method of estimation of treatment efficacy, whereas it would enable performing pre- and post-operative analysis of changes in orbital volume and can serve as one of the criteria for assessing the treatment. However, the implication of this method could be limited to the CT quality, the presence of additional facial pathology, and insufficiency in the orbital volume difference [20].

## 5. Conclusions

Orbital volume, depth, and surface area measurements based on an automated CT segmentation algorithm demonstrated high repeatability and reliability. Male orbits were always larger than female orbits without any overlap. The volume difference was approximately 14%. There were no differences between the left and right orbits. Interestingly, the ratio between volume and surface area was the same between genders and was approximately 0.75. A similar increase in volume as a function of the relative distance from the apex was observed in both men and women.

## Figures and Tables

**Table 1:** *p*-values from normality tests of data using the Shapiro–Wilk test. *p*-values lower than 0.05 indicate that the data cannot be considered normally distributed.

		Left	Right
Male	Volume	0.0665	0.2865
	Depth	0.3821	0.0933
	Area	0.0769	0.0362
Female	volume	0.0231	0.0142
	Depth	0.036	0.2524
	Area	0.0471	0.0234

**Table 2:** The average and median orbital depth, volume, and surface area with their standard deviations and ranges. There were 91 males and 83 females in the study.

		Mean		Median		Std		Min		Max	
		Left	Right	Left	Right	Left	Right	Left	Right	Left	Right
Depth (mm)	Male	39.74	39.83	40.00	40.29	2.91	3.07	31.27	31.24	46.15	46.53
	Female	37.41	37.27	37.33	37.30	2.67	2.65	29.45	29.26	46.06	45.44
Volume (mL)	Male	28.95	29.02	29.61	29.40	3.63	3.63	17.37	17.91	38.43	36.85
	Female	25.39	25.48	25.13	25.19	3.14	3.25	17.36	17.90	35.29	36.78
Area (mm^2^)	Male	3878.68	3871.49	3952.46	3898.07	395.61	382.11	2697.10	2646.76	4648.56	4793.88
	Female	3492.22	3490.72	3465.58	3494.79	339.04	336.55	2712.26	2628.22	4523.00	4448.10

**Table 3:** *p*-values from unpaired *t*-tests for orbital volume, depth, and surface areas. Male and female groups left and right orbits and larger and smaller orbits were studied. In addition to unpaired *t*-tests, paired *t*-tests were also studied for comparing the left and right as well as larger and smaller orbits. The results from paired *t*-tests are presented in round brackets.

	Male vs. Female		Left vs. Right		Larger vs. Smaller	
	Left	Right	Male	Female	Male	Female
Volume	1.00 × 10^−10^	2.00 × 10^−10^	0.90 (0.39)	0.87 (0.25)	0.30 (9.08 × 10^−17^)	0.29 (4.75 × 10^19^)
Depth	4.78 × 10^−8^	2.34 × 10^−8^	0.83 (0.29)	0.74 (0.09)	0.17 (5.47 × 10^−15^)	0.17 (3.11 × 10^−19^)
Area	7.36 × 10^−11^	1.03 × 10^−10^	0.90 (0.47)	0.98 (0.90)	0.23 (4.04 × 10^−16^)	0.18 (3.50 × 10^−11^)

**Table 4:** Correlation coefficients (R^2^) for correlations of orbital volume and depth (V × L), orbital surface area and depth (A × L), and orbital surface area and volume (A × V). Positive correlations were found in both male and female groups.

	Men		Women	
	Left	Right	Left	Right
V × L	0.71	0.73	0.58	0.51
A × L	0.85	0.87	0.84	0.78
A × V	0.96	0.95	0.87	0.87

**Table 5:** *p*-values for parameters in the linear model Volume~1 + Gender + Depth + Gender:Depth. Only depth has a significant effect on the volume. Gender and interaction of gender and depth of the orbit do not have a significant effect on the volume of the orbit.

	Left Orbit	Right Orbit
Gender	0.28	0.36
Depth	7.5 × 10^−21^	3.8 × 10^−19^
Gender:Depth	0.17	0.24

**Table 6:** Intra- and interobserver difference in volume. Example volume for left and right orbits as measured by one of the six operators are presented in the table with the intra- and interoperator variances. Intraoperator variance (ICC_intra_) presents the agreement of two consecutive measurements of orbits from all observers. Interoperator variance (ICC_inter_) presents the agreement of measurements between the six operators for each test case. Both inter- and intraoperator ICCs were excellent (1.0), showing that the automatic segmentation algorithm produces identical segmentation for each image in all the cases.

Case	Left	Right	
	Volume (mL)	Volume (mL)	ICC_Inter_
1	26.2385062253374	26.2451287432629	1.0
2	21.9440769622081	22.0701658712400	1.0
3	24.0008755588866	23.1272418086540	1.0
4	27.2269541561696	27.7177708636990	1.0
5	24.5145413957817	24.4206587669105	1.0
6	32.0841617415446	30.3179772618623	1.0
7	22.1295163397493	22.2934083714752	1.0
8	28.3998038601918	28.6345913023606	1.0
9	24.5112274735212	24.7703571039611	1.0
10	25.5450934482375	25.4178357770615	1.0
ICC_intra_	1.0	1.0	Excellent

## Data Availability

Data available on request from the authors.
